# The multidisciplinary management of hip fractures in older patients

**DOI:** 10.1016/j.mporth.2016.03.006

**Published:** 2016-04

**Authors:** Anna H.K. Riemen, James D. Hutchison

**Affiliations:** **Anna H K Riemen BSc Hon Biochemistry MBChB MRCSEd** Speciality Registrar in Trauma and Orthopaedics, STMTI-Wellcome Trust Clinical Fellow, Regenerative Medicine Group, Arthritis and Musculoskeletal Medicine Programme, University of Aberdeen, Institute of Medical Sciences, Foresterhill, UK. Conflict of interest: none declared; **James D Hutchison PhD FRCSEd FRCSEng FRCSGlas FFSTEd** Regius Chair of Surgery and Sir Harry Platt Chair of Orthopaedic Surgery, Honorary Consultant Orthopaedic Surgeon, Department of Surgery, Medical School, Polwarth Building, Foresterhill, Aberdeen, UK. Conflict of interest: none declared

**Keywords:** Hip fracture, multidisciplinary, occupational therapy, orthogeriatrician, patient centred care, physiotherapy

## Abstract

Older patients presenting with hip fractures are some of the frailest and sickest patients in hospital. In addition to complex medical problems and comorbidities, they have to overcome the additional physiological challenges posed by the hip fracture itself, and subsequent surgery. Hip fracture associated morbidity and mortality at one year remains high. Published guidelines stress the need for a multidisciplinary approach and the importance of the care environment for good outcomes. A combined management approach identifies and addresses not only the surgical but also the complex analgesic, medical, cognitive, nutritional, social and rehabilitation needs of our patients, thereby improving outcome for our patients.

## Introduction

The lifetime risk of sustaining a hip fracture in the United Kingdom from age 50 is around 11% for women and 3% for men.^1^ Hip fractures have a devastating impact on patients including death, depression, disability, institutionalisation, fear of falling, and social isolation.^2^, ^3^ Older patients presenting with hip fractures comprise some of the frailest and sickest patients, with complex medical problems and comorbidities, who have to overcome the additional physiological challenges posed by trauma and surgery.^4^ Consequently, hip fracture associated morbidity and mortality remains high, with approximately 10% of patients dying within 1 month, 30% at 1 year and 80% at 8 years following hip fracture.^5^, ^6^, ^7^ Death tends to be associated with a patient's comorbidities, rather than the hip fracture itself. Nearly 40% of patients will not return to their pre injury residence.^8^

Published guidelines stress the need for a multidisciplinary approach and the importance of the care environment for good outcomes. The Scottish Intercollegiate Guidelines Network (SIGN), the National Institute for Clinical Excellence (NICE), and the British Orthopaedic Association in cooperation with the British Geriatric Society, have all produced guidelines supporting a multidisciplinary team approach and stress the need for inclusion of a geriatrician from the time of admission.^9^, ^10^, ^11^ Simple steps, such as a formal falls assessment, have been shown to reduce morbidity and mortality in hip fracture patients.^9^, ^10^, ^11^, ^12^, ^13^, ^14^

A combined management approach (Box 1) identifies and addresses not only the surgical but also the complex analgesic, medical, cognitive, nutritional, social and rehabilitation needs of these patients, with concomitantly improved outcomes.^8^ A recent Cochrane review found that older patients, who are part of enhanced multidisciplinary care and rehabilitation models, had lower complications rates, reduced length of hospital stay and institutional placement, as well as better function and achievement of pre-injury walking ability.^15^

**Figure 1:**
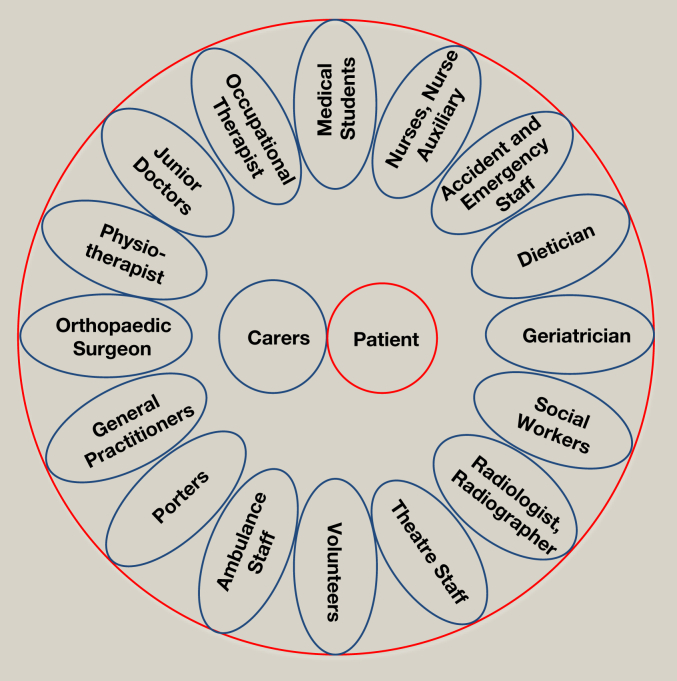
The multidisciplinary team involved in looking after hip fracture patients is extensive.

It is recognized that a team approach with excellent communication between all the members is essential. The multidisciplinary team looking after hip fracture patients is large (Figure 1), and each role is important in the jigsaw of care.

### Accident and emergency (A&E)

At the start of the patient's journey through the hospital system, their initial management lays the foundation for subsequent care. Rapid assessment and first line investigations for patients suffering a hip fracture can identify other injuries and medical conditions early, allowing timely optimisation for surgery.^4^ The “Scottish Standards of Care for Hip Fracture Patients” records each hospital's performance, aiming for 100% of hip fracture patients attending A&E to have all of the “Big Six” interventions carried out – analgesia prescribed, NEWS score recorded, pressure areas assessed, intravenous/oral fluids prescribed (as clinically appropriate), bloods taken, and cognition screening performed. A structured hip fracture pathway can provide a tool to achieve this, prompting every member of the team, and allowing for documentation to avoid duplication. Several hospitals have also successfully introduced fast-tracking of hip fracture patients through A&E; however it is important that good clinical care should not be prejudiced by an administrative “tick box” drive to achieve an arbitrary standard.

### Nurses, nurse auxiliary and advanced nurse practitioners

Nurses have a key and essential role in providing the care for these complex patients. Nurses are uniquely placed to spend time communicating with the patient and carers and finding out about a patient's pre-injury or pre-confusion state.

Pressure care is essential and ward nurses will continue and expand on the initial care delivered. Patients may already have a pressure area problem from prolonged lying on the floor following a fall, may be malnourished and/or dehydrated, and have pre-existing poor mobility – all leading to increased risk.

Hip fracture patients often do not achieve their required nutritional intake. Poor nutrition is a risk factor for poor wound and fracture healing. Prolonged repeated fasting times can be detrimental to health and rehabilitation, and nursing staff are excellently placed to liaise with surgical and anaesthetic staff to minimize pre-operative fasting times.

Older patients admitted to hospital often undergo a functional decline due to reduced physical activity. This is in part due to the injury, but can also be due to enforced bed rest as a result of devices and interventions tethering patient to their bed. In acute trauma wards there is often limited opportunity for physical activity. However, when nurses encourage patients to remain physically active and participate in self care, this decline is reduced.^16^, ^17^

The introduction in recent years of specialist nurses to look after older hip fracture patients has proved to be very helpful, promoting sustainable high standards of care. They support the geriatricians (*vide infra*) and provide a mechanism for holistic and regular review of these frail older patients, as well as providing a vital link to family and carers.

Geriatric or hip fracture liaison nurses can provide a link to other specialities and are invaluable in providing regular geriatric input for trauma wards and *vice versa*. They coordinate and accompany the patient's journey of care, liaising with other specialities, facilitating rehabilitation, discharge and follow up planning. Having a member of staff who provides continuity and communication with everyone including the family is invaluable, particularly in an era of junior doctor shift work and the loss of the medical team structure.^4^

### Physiotherapy

All guidelines recommend physiotherapy assessment and mobilisation on the first day following surgery and then at least once daily.^6^ The goal of operative treatment of hip fractures is to enable immediate weight bearing without restriction, facilitating early physiotherapy assessment and intervention.^8^ Inability of the patient to undertake a physiotherapy programme on the first postoperative day is a strong predictor for not regaining basic mobility on discharge.^18^

While the physiotherapist concentrates on strengthening, range of movement and gait training exercises, evidenced pathways for management following the different surgical interventions following hip fracture are lacking, and warrant further research.

In studies involving intensive physiotherapy, functional outcomes were better at discharge,^19^ and in combination with early surgery and mobilisation, resulted in shorter hospital stays.^20^ However, in the longer term there was little difference between standard care and enhanced intensive physiotherapy regimes, although the studies do not measure whether there was a difference in quality of life or patient satisfaction resulting from an earlier return to mobility. Strength and mobility scores were found to be better in groups of patients receiving quadriceps strengthening exercises with physiotherapy 20 minutes a day, five days a week for six weeks.^21^ Continued home programmes combining physiotherapy with occupational therapy (focused on activities of daily living) result in improved balance, strength and mobility at six months. Similar results are achieved with aerobic exercise programmes in the community. A systematic review by Chudyk et al. found focussed exercise programmes to deliver functional improvement at three and six months, with any advantage disappearing by one year.^22^

A physiotherapist's role is wider than physical rehabilitation of the patient; through their interaction with patients and carers, they can undertake other aspects of care such as being Dementia Champions, and are key in discharge planning.

They are also well placed to contribute to hip fracture prevention through interventions such as falls groups and balance classes, for example by targeting wrist fracture patients to intervene before a hip fracture occurs.

### Occupational therapy

Occupational Therapists in our unit work closely with their physiotherapist colleagues to assess and educate patients regarding safety with transferring, washing and self care. When needed they can provide aids or organize home modifications to facilitate safety and independence at home. Ideally, the occupational therapist should visit and assess patients in their own home. When it is not possible, they have to rely on relatives for information, such as the height of furniture at home, and perform assessments such as a kitchen assessment in the hospital. One traditional aspect, the role of providing patients with an “occupation” during their hospital or rehabilitation time, has long since disappeared. Staffing levels and infection control measures limit the options available on how to occupy the patient's time in hospital. Despite these restrictions, some more recent initiatives, such as the use of therapy pets, have proved very popular with patients.

### Dietician

As mentioned above, the nutritional status of hip fracture patients can often be poor at the time of admission and can decline thereafter for various reasons, such as repeated pre-operative fasting, during the hospital stay.^23^, ^24^ Poor nutritional status worsens the age-related decline in muscle mass, and is associated with an increased risk of complications and functional decline.^23^, ^24^, ^25^, ^26^, ^27^ In a recent study on voluntary dietary intake and the nutritional status of hip fracture patients in hospital, patients with a low intake had more complications such as infection and were more likely to be in a care facility four months after the injury.^28^ It is therefore essential that hip fracture patients are assessed on admission, using a simple tool such as the Malnutrition Universal Screening Tool (MUST score)^29^ and are offered interventions to improve nutritional intake. This can involve simple steps such as minimising preoperative fasting times, encouraging relatives to bring in favourite foods and tailored appetising meal options.

### Patient

Just like any other patient, those with hip fractures should be involved in their care and have the opportunity to make informed decisions about their care and treatment. They are at the centre of the multidisciplinary team. The NICE guidelines recommend good quality written evidence-based materials tailored to specific needs of patients and their carers. Such information should explain the diagnosis of hip fracture, choice of anaesthesia, analgesia and other medication, surgical procedures, possible complications, postoperative care, rehabilitation programme, long term outcomes, as well as the healthcare professionals involved in their care, including their roles.^6^ Much attention has recently been focussed on the “responsible clinician”, and the name of the consultant should be clearly displayed above the patient's bed.^30^ In addition to the physical injury, patients suffer from a loss of confidence and fear of falling following a hip fracture. To minimize future falls risk, patients should undergo a multifactorial falls assessment (Box 2).

### Carers

Carers are an easily forgotten group, but they are the patient's support network with the capacity to enhance patient care though practical help and psychosocial support, aiding recovery. Carers can be a valuable resource for hospital staff, with key information on the patient's pre-fracture status (physical and mental) and their likes and dislikes.^31^ Indeed there are strategy documents that recognize the importance of the carers as partners in looking after patients.^31^ Carers for hip fracture patients are mostly first and second degree relatives, a third of whom are in employment and over half live a significant distance away. During hospital visits they help with eating by providing special foods, encouraging food and fluid intake. Carers provide social interaction such as going through mail, banking, conversation and looking at a newspaper, thereby linking the patient to external world. They are a source of emotional support by providing reassurance and encouragement. Communication with carers can easily be missed and they often rely on getting information through indirect means or through assumptions. There is an unmet need for more information, creating anxiety on the carer's part.^8^, ^31^

The introduction of open visiting times and an increased effort for communication with carers are positive changes; communication with the patient and their carer needs to be open and realistic.^8^

### Geriatrician

Patterns of orthogeriatric care vary between hospitals, from traditional orthopaedic care with variable geriatric input, to combined orthogeriatric care, or even geriatric care with surgical input. In all guidelines it is recognized that geriatric input should ideally start from admission and continue at regular intervals.^4^, ^6^, ^10^

Studies assessing joint care scenarios between geriatricians and orthopaedic surgeons are limited, but there is evidence that they can result in better functional outcomes at discharge and three months follow up, although no difference was evident beyond six months. Similar results can be achieved in patients with dementia in whom joint care results in higher independence at three months.^22^

In models where a hip fracture is considered a geriatric problem, with surgery to “fix the fracture” being an essential but overall small aspect of care, the outcomes surpass those where fracture fixation alone is the primary focus. This has long been reflected in clinical guidelines but implementation of this concept into daily practice and culture is an ongoing process.^4^, ^6^, ^9^, ^10^, ^11^ In a randomized controlled trial with very well matched groups of patients, who lived independently prior to sustaining a hip fracture, more patients were discharged directly home from comprehensive geriatric care compared to orthopaedic care, at a cost of 1.6 additional hospital days. Those patients had better mobility and scored higher in quality of life and cognitive scores. Subsequent cost from rehabilitation, nursing home stay and further hospital stays were higher in the standard orthopaedic care group, although this did not reach statistically significant levels.^32^ In general, however, audit has consistently shown that hospitals which routinely transfer hip fracture patients to rehabilitation units have longer in-patient stays than those hospitals who discharge their hip fracture patients directly from the acute orthopaedic ward. Another argument against “hip fracture wards” is that hip fractures represent only about 15% of fragility fractures, and there are many other frail older patients who require the same level of medical input and care.

With orthogeriatricians involved in the care of patients on the acute trauma wards, issues such as osteoporosis, medication review and falls prevention can be addressed early and included in the rehabilitation planning.^4^ Early medical review on admission should also facilitate rapid optimisation for surgery, although there have been reports that it can delay surgery if extensive medical investigations are requested pre-operatively. There is no evidence to support delaying surgery to await investigations such as echocardiography or to optimize anaemia, anticoagulation, uncontrolled heart failure or correctable arrhythmias beyond 24 hours from admission.^8^

### Foundation doctor

The most junior member of the medical team, located on the wards, spends significant amounts of time with the patient. While foundation doctors are valuable team members (where teams still exist), they also gain from this experience. There has been a suggestion that admitting and looking after these patients is “repetitive and not educationally useful”; this could not be further from the truth and if junior doctors think this to be the case, they should question why they are doing medicine in the first place. They learn how to look after frail sick patients who have significant injuries and the physiological challenge of surgery on top of their complex medical needs. The problems for junior doctors now is the loss of the supportive team structure in modern British hospital medicine, and the large numbers of sick patients that they have to look after overnight.

### Primary care

General Practitioners are another easily forgotten resource. They know the patient before their injury and will continue to care for them once discharged back into the community. Communication is a key, and the availability of the electronic Emergency Care Summary Record on admission of the patient has been an important development.^33^ Similarly, on discharge, clear and accurate summaries are vital for patient's transition back into the community. To avoid duplication or errors of failed follow up, it is essential to include clear information on specialist reviews, investigation and management recommendations.

## Clinical pathways

Clinical pathways are designed to facilitate multidisciplinary team working. Through a description of the expected interventions and outcomes along the patient journey following a hip fracture, everyone knows the next step; unnecessary variations in practice can be avoided.^22^

### Early supported discharge

Many units now have discharge coordinators and early supported discharge teams. Often these are invaluable points of contact with families.^31^ Discharge coordinators strive to achieve a seamless transition and progression in the patient journey, but may be constrained by the availability of care and rehabilitation facilities, equipment, district nurses and ultimately theatre space. If any of these are lacking, the patient's journey is halted. It should be borne in mind however that a quick, early discharge home may not always be in the best interest of the patient; a study on length of hospital stay after hip fracture in Sweden showed a higher 30-day mortality rate with a length of stay of 10 days or less.^34^

### Falls prevention programmes

NICE Guideline 21 recommends that “older people who present to medical attention because of a fall … should be offered a multifactorial falls risk assessment”. These come in a variety of forms but all have essentially a similar set of components many of which could form part of the initial assessment and hospital stay of a hip fracture patient (Box 2). In our unit we developed a pro-forma for the admission of hip fracture patients, greatly improving our documentation of many of the components of falls assessments. In successful multifactorial intervention programmes the common components include strength and balance training, assessment of home hazards, an assessment of vision with appropriate referral, and a review of medication with modification or withdrawal.

### Measuring good multidisciplinary team performance

Scotland conducted an audit of hips fracture care from 1993 to 2008. The synergy between an evidence-based guideline of best practice (SIGN Guidelines 15, 56, 111) and the Scottish Hip Fracture Audit resulted in improvement of care across the board. For example, the time from admission to theatre improved to less than 48 hours for 98% of patients across Scotland, having been as low as 15% in some units. Routine data collection stopped in 2008 as funding was transferred to a more generalized musculoskeletal audit.^14^ Within this, data have been collected again on a rolling basis for hip fracture patients, and again this has shown how audit has helped to improve standards. In England the National Hip Fracture Database (NHFD), established in 2007, collects and reports on what is now the largest set of hip fracture records in the world.^35^ Not only are outcomes measured but Hospital Trusts are also visited and recommendations made for improvement.

A measure for good multidisciplinary team work is a trauma meeting in which the whole multidisciplinary team is not only present but also interacts.^36^ Indeed, hip fractures are a tracer condition which tests not only different specialities, professions, institutions and agencies but also how well they work together – or not.

## Conclusion

Ultimately, patient-centred multidisciplinary care, extending beyond the hospital setting, provides the best outcomes and measures healthcare effectiveness beyond just the care of hip fracture patients.^4^ While we have now recognized that good multidisciplinary team work and care is essential for these patients, aspects of interventions undertaken throughout the care pathway need a more structured assessment though randomized controlled trials.^6^, ^15^

**Learning points**

• Older patients admitted with hips fractures are some of the frailest and sickest patient in the hospital requiring a multidisciplinary approach
• Good and open communication between the members of the multidisciplinary team is essential
• Care for hip fracture patients is a surrogate marker on how hospitals deal with frail, older patients

## Box 1 A multidisciplinary hip fracture programme has seven components

**Components of a multidisciplinary hip fracture programme**^6^, ^8^
• Orthogeriatric assessment
• Rapid optimisation of fitness for surgery
• Early identification of individual goals for multidisciplinary rehabilitation
• Continued, coordinated orthogeriatric and multidisciplinary review
• Liaison with other services (mental health, falls prevention, bone health, primary care, social services)
• Governance structure for all stages
• Palliative care (if fracture due or triggers terminal illness)

## Box 2 Some of the components of falls assessments

**Components of falls assessments**^9^
• Identification of falls history
• Cardiovascular examination
• Neurological examination
• Gait, balance, mobility, muscle weakness
• Cognitive impairment
• Visual impairment
• Medication review
• Osteoporosis risk
• Perceived functional ability
• Fear related to falling
• Continence
• Home hazards

## References

[1] van Staa T.P., Dennison E.M., Leufkens H.G., Cooper C. (2001). Epidemiology of fractures in England and Wales. Bone.

[2] Volpato S., Guralnik J.M. (2015). Hip fractures: comprehensive geriatric care and recovery. Lancet.

[3] Magaziner J., Hawkes W., Hebel J.R. (2000). Recovery from hip fracture in eight areas of function. J Gerontol A Biol Sci Med Sci.

[4] British Orthopaedic Association (2007). The care of patients with fragility fracture.

[5] Bentler S.E., Liu L., Obrizan M. (2009). The aftermath of hip fracture: discharge placement, functional status change, and mortality. Am J Epidemiol.

[6] National Institute for Health and Clinical Excellence, The management of hip fracture in adults. Clinical guidline 2011, London: National Institute for Health and Clinical Excellence.

[7] Johnston A.T., Barnsdale L., Smith R., Duncan K., Hutchison J.D. (2010). Change in long-term mortality associated with fractures of the hip: evidence from the scottish hip fracture audit. J Bone Jt Surg Br.

[8] Chesser T., Kelly M. (2013). Management of hip fractures in the elderly. Surg – Oxf Int Ed.

[9] National Institute for Clinical Excellence, Falls: the assessment and prevention of falls in older people. Clinical guideline 21. London: National Institute for Clinical Excellence.

[10] Scottish Intercollegiate Guidelines Network (2009). Management of hip fracture in older people: a national clinical guideline.

[11] Standards for Trauma, British Orthopaedic Association (2012). BOAST 1-Patients sustaining a fragility hip fracture.

[12] Mayor S. (2012). Length of hospital stay for hip fracture falls by a day, audit shows. BMJ.

[13] White C. (2010). UK hip fracture audit shows wide variation in standards of care. BMJ.

[14] Currie C., Partridge M., Plant F., Roberts J., Wakeman R., Williams A. (2012). The national hip fracture database national report.

[15] Smith T.O., Hameed Y.A., Cross J.L., Henderson C., Sahota O., Fox C. (2015). Enhanced rehabilitation and care models for adults with dementia following hip fracture surgery. Cochrane Database Syst Rev.

[16] Resnick B., Galik E., Wells C.L., Boltz M., Holtzman L. (2015). Optimizing physical activity among older adults post trauma: overcoming system and patient challenges. Int J Orthop Trauma Nurs.

[17] Boltz M., Resnick B., Capezuti E., Shuluk J. (2014). Activity restriction vs. self-direction: hospitalised older adults' response to fear of falling. Int J Older People Nurs.

[18] Hulsbaek S., Larsen R.F., Troelsen A. (2015). Predictors of not regaining basic mobility after hip fracture surgery. Disabil Rehabil.

[19] Swanson C.E., Day G.A., Yelland C.E. (1998). The management of elderly patients with femoral fractures. A randomised controlled trial of early intervention versus standard care. Med J Aust.

[20] Koval K.J., Aharonoff G.B., Su E.T., Zuckerman J.D. (1998). Effect of acute inpatient rehabilitation on outcome after fracture of the femoral neck or intertrochanteric fracture. J Bone Jt Surg Am.

[21] Mitchell S.L., Stott D.J., Martin B.J., Grant S.J. (2001). Randomized controlled trial of quadriceps training after proximal femoral fracture. Clin Rehabil.

[22] Chudyk A.M., Jutai J.W., Petrella R.J., Speechley M. (2009). Systematic review of hip fracture rehabilitation practices in the elderly. Arch Phys Med Rehabil.

[23] Koren-Hakim T., Weiss A., Hershkovitz A. (2012). The relationship between nutritional status of hip fracture operated elderly patients and their functioning, comorbidity and outcome. Clin Nutr.

[24] Corish C.A., Kennedy N.P. (2000). Protein-energy undernutrition in hospital in-patients. Br J Nutr.

[25] Nuotio M., Luukkaala T. (2015). Factors associated with changes in mobility and living arrangements in a comprehensive geriatric outpatient assessment after hip fracture. Disabil Rehabil.

[26] Nuotio M., Tuominen P., Luukkaala T. (2015). Association of nutritional status as measured by the Mini-Nutritional Assessment Short Form with changes in mobility, institutionalization and death after hip fracture. Eur J Clin Nutr.

[27] Pajulammi H.M., Pihlajamaki H.K., Luukkaala T.H., Nuotio M.S. (2015). Pre- and perioperative predictors of changes in mobility and living arrangements after hip fracture–a population-based study. Arch Gerontol Geriatr.

[28] Goisser S., Schrader E., Singler K. (2015). Low postoperative dietary intake is associated with worse functional course in geriatric patients up to 6 months after hip fracture. Br J Nutr.

[29] Stratton R.J., King C.L., Stroud M.A., Jackson A.A., Elia M. (2006). ‘Malnutrition Universal Screening Tool’ predicts mortality and length of hospital stay in acutely ill elderly. Br J Nutr.

[30] Francis R., Mid Staffordshire NHS Foundation Trust Public Inquiry (2013). Report of the mid Staffordshire NHS foundation trust public inquiry. Hc [2012–13].

[31] Macleod M., Chesson R.A., Blackledge P., Hutchison J.D., Ruta N. (2005). To what extent are carers involved in the care and rehabilitation of patients with hip fracture?. Disabil Rehabil.

[32] Prestmo A., Hagen G., Sletvold O. (2015). Comprehensive geriatric care for patients with hip fractures: a prospective, randomised, controlled trial. Lancet.

[33] Morris L.M., Brown C., Williamson M., Wyatt J.C. (2012). The Scottish Emergency Care Summary–an evaluation of a national shared record system aiming to improve patient care: technology report. Inf Prim Care.

[34] Nordstrom P., Gustafson Y., Michaelsson K., Nordstrom A. (2015). Length of hospital stay after hip fracture and short term risk of death after discharge: a total cohort study in Sweden. BMJ.

[35] Chesser T., Dixon P., Wilson H., Hertz K., Moppett I. (2015). The BOA multidisciplinary hip fracture reviews. J Trauma & Orthop.

[36] Wakeman R., Boulton C. (2016). Lessons from the national hip fracture database. Orthop Trauma.

